# Observational evidence of egg guarding in wild European squid

**DOI:** 10.1002/ece3.70111

**Published:** 2024-08-07

**Authors:** Jorge Hernández‐Urcera, Ángel F. González, Felipe Escolano, Miguel Cabanellas‐Reboredo

**Affiliations:** ^1^ ECOBIOMAR Research Group Institute of Marine Research (IIM‐CSIC) Vigo Spain; ^2^ National Center Spanish Institute of Oceanography, CSIC Balearic Islands CO Spain

**Keywords:** cephalopods, egg care, egg masses, *Loligo vulgaris*, squid

## Abstract

It is accepted that loliginids, like other squid, deposit their eggs in crevices on the seabed and then abandon them. In this work, we present observational evidence of egg guarding behavior in wild European squid, *Loligo vulgaris*. While monitoring a squid spawning crevice at night in Spain, a large mass of squid eggs was located and filmed 17 times during 42 days, until hatching. A male and a female of *L. vulgaris* were filmed in front of the crevice. The same male was filmed guarding the eggs on consecutive days. In the presence of the divers, male and female alternated their approaches to the crevice repeatedly touching and flushing the egg clusters. This guarding behavior differs from the reproductive habits assumed for the European squid and could represent the first evidence of egg guarding by a male in cephalopods.

## Graphical abstract

In this work, we present observational evidence of egg guarding behavior in wild European squid, *Loligo vulgaris*. Our finding is unexpected because this behavior differs from the reproductive habits assumed for this species. This behavior could represent the first evidence of egg care by a male in cephalopods.

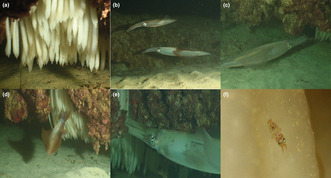

## 1 INTRODUCTION

The term “egg care” in marine invertebrates refers to the process by which these organisms provide care and protection to their eggs. This can involve various behaviors and strategies aimed at ensuring the survival and development of the offspring, such as guarding the eggs, cleaning them, aerating them, or providing them with nutrients (Stahlschmidt & DeNardo, 2011). Egg care is taxonomically widespread and is central to the maintenance of biodiversity through its close association with other phenomena such as sexual selection, life‐history evolution, sociality, genetic architecture, and phenotypic plasticity (Royle et al., 2012). In cephalopods, it was initially assumed that the sole form of egg care is exhibited by some females, with no evidence shown for males (Hanlon & Messenger, 2018). To date, in loliginid squid, it was accepted that they select optimal spatio‐temporal windows of oceanographic and biological conditions, where they deposit their eggs and leave them with no signs of parental care (Boavida‐Portugal et al., 2010; Hanlon & Messenger, 2018; Villanueva et al., 2003).

The European squid (*Loligo vulgaris*) is an important resource from an ecological and socioecological perspective. It is targeted by some commercial and recreational fisheries in Europe (Cabanellas‐Reboredo et al., 2017; Pierce et al., 2010). It plays an essential role in ecosystem functioning due to its central trophic web position (Valls et al., 2015). However, despite the importance, some aspects of its behavior, such as mating and breeding, are poorly understood (Hanlon & Messenger, 2018), with merely anecdotic reports of its reproductive behavior (Cabanellas‐Reboredo et al., 2012, 2014). *L. vulgaris* females have been reported to lay eggs in clusters (egg masses) attached to different hard substrates or branched sessile organisms (Jereb & Roper, 2010). Egg masses consist of numerous finger‐like capsules that females fasten to the selected substrate one by one. Finding egg masses in the wild appears to be a major challenge (Cabanellas‐Reboredo et al., 2014); thus, a complete monitoring of egg masses in the wild has not been reported. However, it is assumed that, after spawning, the eggs develop on their own without further contact with their parents (Hanlon & Messenger, 2018). In this study, after monitoring egg masses, from laying to hatching, through in situ observations in the wild, evidence of egg guarding in *L. vulgaris* has been observed.

## 2 MATERIALS AND METHODS

A spawning crevice of *L. vulgaris* was located on the Spanish Mediterranean coast at 7 m depth (Jávea, 38°47′54″ N 0°11′22″ E). It was monitored at night between December 2018 and February 2019 (Table 1). This crevice was chosen for monitoring because egg masses of *L. vulgaris* had been found there in previous years (F. Escolano, personal observation). The start time of each observation and the water temperature were automatically recorded by a dive computer. Egg masses and other notable occurrences in the spawning area, such as the presence of adult squid and visual displays, were recorded using a Sony HVR A‐1 camera in an underwater housing. The total number of egg strings in the egg masses was estimated from the video images using the Image J software. The sex and identification of the adults were determined through their mantle patterns, analyzing the lateral mantle streaks (LMS). LMS are essentially male‐only streaks of chromatophores. Large mature males have 8–17 streaks, whereas mature females have only 1–3, often quite small and indistinct (Hanlon et al., 1994). A sample of eggs was collected for genetic analysis of the partial COI sequences using the MEGA10 software.

**TABLE 1:** Observational data from the spawning crevice.

Date	Time	*T*	DE	Observations
Dec‐19	19:15	13.2	–	Empty
Dec‐23	18:45	13.2	–	Empty
Jan‐2	18:30	13.4	–	Empty
Jan‐6	18:45	13.5	0	Egg masses
Jan‐7	18:30	13.4	1	Egg masses
Jan‐8	19:00	13.3	2	Egg masses +1 squid (aggressive male)
Jan‐9	18:30	13.4	3	Egg masses
Jan‐11	20:15	13.3	5	Egg masses
Jan‐14	21:45	13.3	8	Egg masses +2 squid (guarding)
Jan‐15	20:45	13.2	9	Egg masses +2 squid (guarding, M1 and female)
Jan‐16	08:45	13.2	10	Egg masses +1 squid (guarding, M1)
Jan‐16	19:45	13.2	10	Egg masses
Jan‐18	20:30	13.3	12	Egg masses
Jan‐20	21:00	13.2	14	Egg masses
Jan‐22	20:30	13.3	16	Egg masses
Feb‐5	19:45	13.3	30	Egg masses
Feb‐9	20:00	13.2	34	Egg masses
Feb‐12	20:45	13.4	37	Hatching + egg masses
Feb‐13	20:30	13.4	38	Hatching + egg masses
Feb‐19	21:15	13.5	42	Hatching + egg masses

*Note*: Depth: 7 m.

Abbreviations: DE, days elapsed since egg masses were found in the crevice; M1, same identified male; T, temperature; Time, start time of the dive.

## 3 RESULTS

**FIGURE 1:**
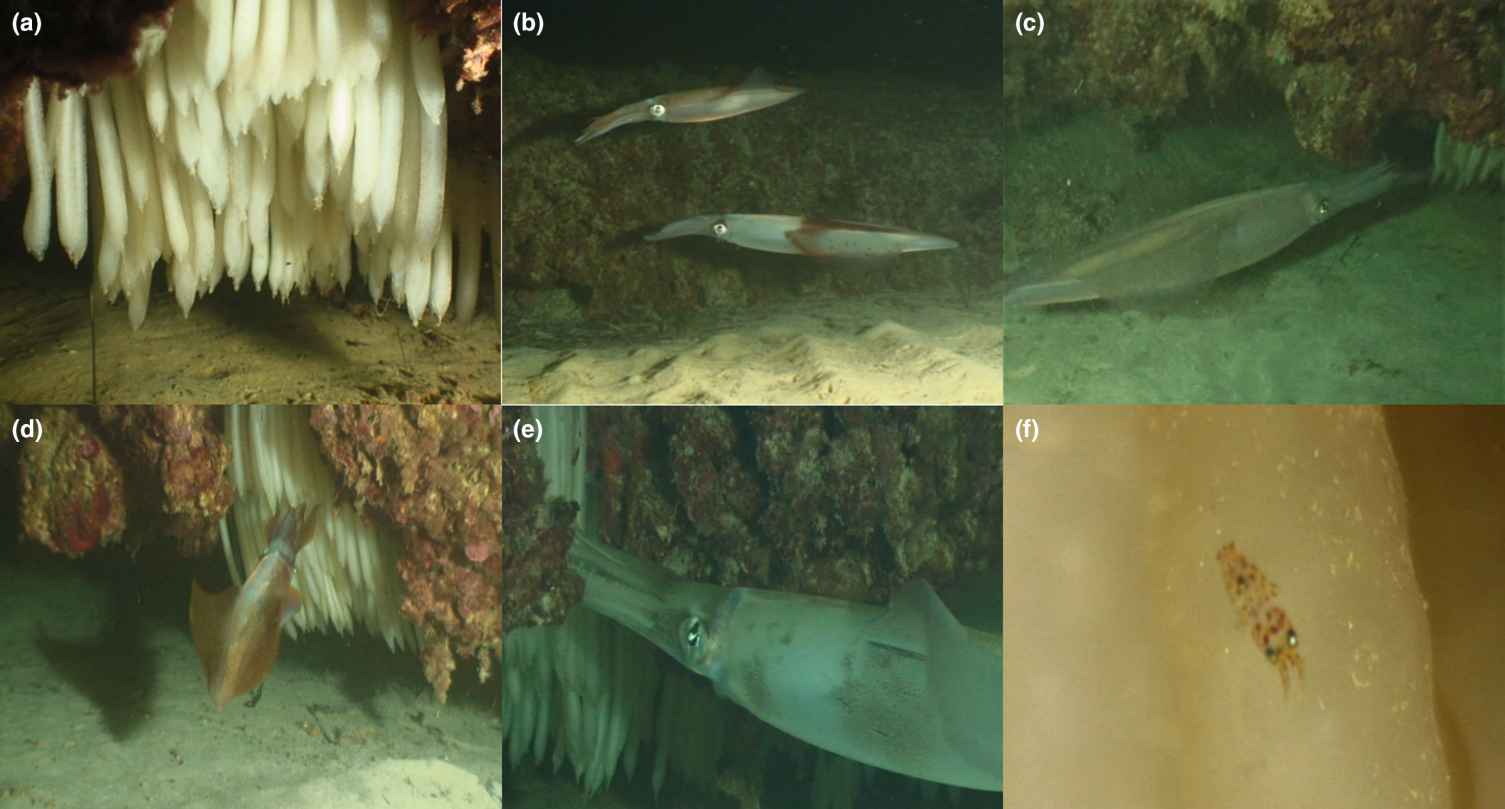
Main behavioral observations (see also Videos S1‐S5). (a) Egg masses in the crevice. (b) A male and a female squid approaching the egg masses. (c) The male touching the strings (Jan‐15). (d) The female touching the strings. (e) The male observed the previous day touching the strings (Jan‐16). (f) First hatchlings.

**FIGURE 2:**
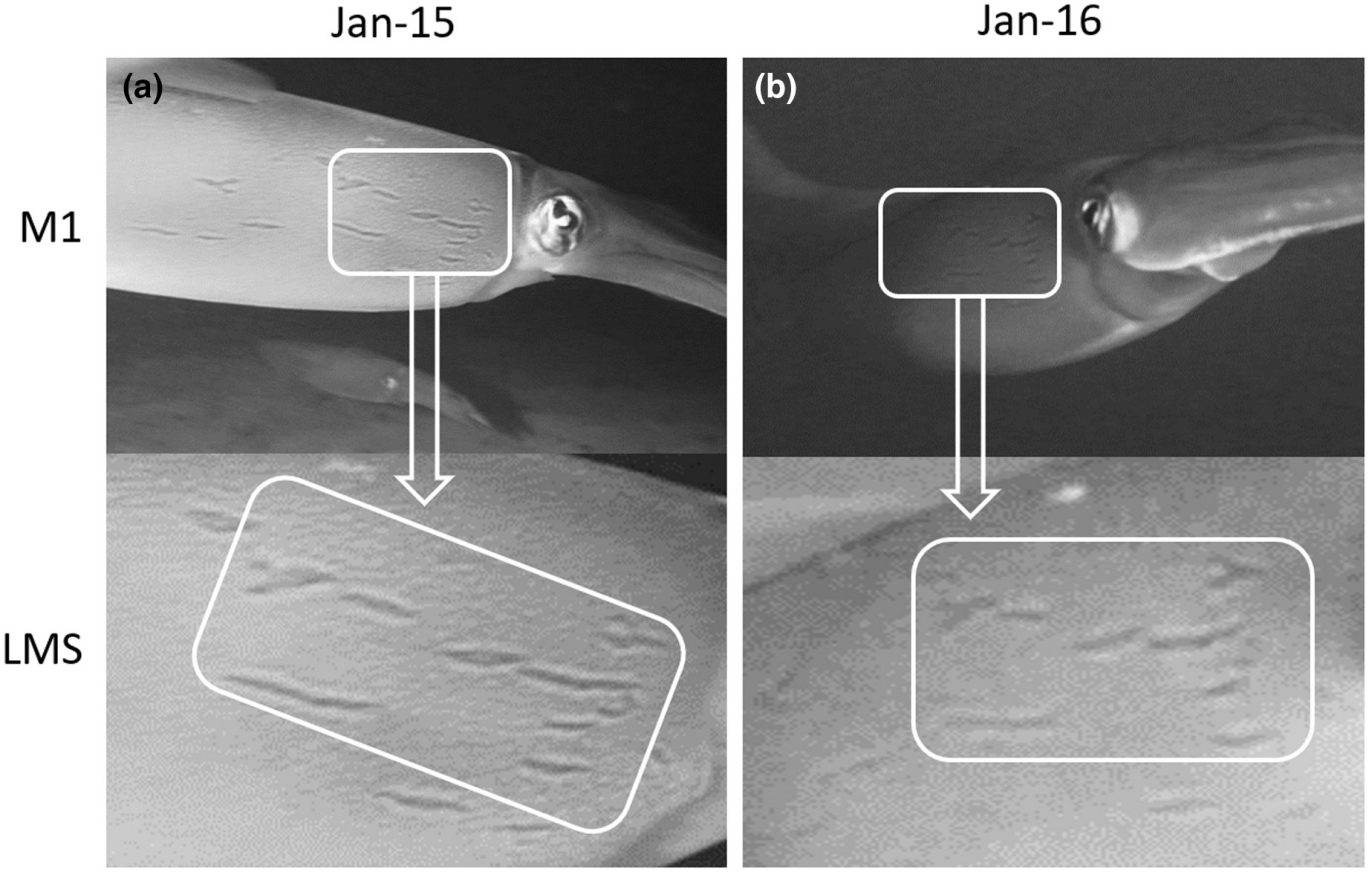
Lateral mantle streaks (LMS) and match pattern (white boxes) observed in the male M1 (*Loligo vulgaris*) on two consecutive days.

The first egg masses were observed 17 days after the crevice started to be monitored (Table 1; Figure 1a; Video S1). Two days later, an adult male near the spawning crevice was recorded, which showed agonistic behavior, even charging at one of the divers (Video S1). Six days later, two adults (male and female) were observed within the proximity of the crevice (Figure 1b; Video S2). They quickly escaped, avoiding recording the LMS patterns. The following night, two adults (male and female) approaching the egg masses were recorded again. A few seconds later, both squid performed a behavior of what appeared to be cleaning operations, repeatedly touching and flushing the egg clusters (Figures 1c and 2d; Video S3). When performing this behavior, male and female took turns, and while one of them touched the eggs, the other remained near the crevice entrance slightly behind its mate (Video S3). The male (M1) had a characteristic lateral mantle streak arrangement (Figure 2a). The next morning (12 h later), an adult male performing the same *touching behavior* on the eggs was founded (Figure 1e, Video S4). Surprisingly, the male was M1 (Figure 2b), and this time it was doing the same behavior alone. Unfortunately, this was the last time when an adult squid was observed near the crevice, being the last record of this behavior. Mean water temperature during monitoring was 13.3 ± 0.1°C. The total number of egg strings was 140, which became increasingly dark as embryonic development progressed. The first hatchlings were observed at day 37 (Table 1; Figure 1f; Video S5). The sequence alignment analysis of the partial COI sequences revealed that all embryos belonged to *Loligo vulgaris* (BLAST ≥ 98% *L. vulgaris*).

## 4 DISCUSSION AND CONCLUSIONS

In *Loligo vulgaris*, social interactions during spawning are apparently limited to females, which, in the presence of laid egg strings, add their own to those previously fixed on the substratum (Laptikhovsky et al., 2021). Egg capsules of the same egg mass might be at different stages of embryonic development because they were laid by different females at different times (Laptikhovsky et al., 2021). Therefore, the egg masses found in the crevice could be laid by more than one female. After spawning, it was assumed that *L. vulgaris* do not care for their eggs, and egg masses are attached to a substratum and remain there for a long period, exposing them to the risk of predation (Villanueva et al., 2003). In this regard, it has been reported that there are low levels of predation on loliginid eggs (Sauer & Smale, 1991), likely due to toxic compounds present in the egg capsules of loliginids produced by bacteria from the female's accessory nidamental glands (Pichon et al., 2005). However, several families of polychaetes have been observed in egg masses of *L. vulgaris* eating the gelatinous mass, surrounding the structure, and making holes, which are used by nematodes, copepods, and ciliates (von Boletzky & Dohle, 1967). In addition, predation by some species of sea stars, sea urchins, and sparid fish on egg masses of loliginids has also been reported (Hixon, 1983; Sauer & Smale, 1993). Despite the risk of predation, it is only known that some oceanic squid display egg care during embryonic development. Brooding deep‐sea female squid hold their eggs with their arms for a period of 6–9 months (Seibel et al., 2005).

Laboratory experiments showed that in other squid species, whose deposit eggs in massive communal spawning arenas and where hundreds of individuals mate, some males increase their aggressiveness when they touch the egg capsules (King et al., 2003). Cummins et al. (2011) reported that *Doryteuthis pealeii* egg capsules contain a factor that causes extreme aggression between males, even in the absence of females. Males were attracted to the eggs visually, but upon touching them and contacting a pheromone synthesized by females, they immediately escalated into intense physical fighting with any nearby male. In *Loligo vulgaris* there are no large aggregations of individuals for mating (Boavida‐Portugal et al., 2010), so situations of agonistic behavior for mating are probably very rare. During our observations, there was never more than one male at the same time in the vicinity of the spawning crevice, so an increase in aggression after touching the eggs could not be recorded. Interestingly, Tardent (1962) described a type “grooming behavior” in captive males of *L. vulgaris*, which was speculated to be associated with cleaning the egg masses (i.e., parental care). Other authors have also documented some form of parental care or guarding behavior in captive males of *L. pealeii* (Stevenson, 1934) and *L. opalescens* (Hurley, 1977). Our observations could confirm this behavior in *L. vulgaris*, providing contemporary evidence in the wild that reinforces the parental care theory.

Furthermore, in our study also females of *L. vulgaris* were found guarding the eggs. Some studies have reported egg touching behaviors by females of *Doryteuthis pealeii* and *D. plei* (Arnold, 1962; Roper, 1965). *D. pealeii* females inspected and touched the eggs within 20 s after the introduction of an egg mass in the tank (Arnold, 1962), and *D. plei* females touched and pumped water through the funnel to the eggs during the first day after laying. In our observations of egg guarding by females, more than 7 days had passed since the egg mass was found. In addition, the experiments with *Dorytheutis* sp. have been conducted in laboratory settings with captive squid where the animals were in close proximity to egg masses due to space limitations in the tanks. This spatial confinement may lead to observations of behaviors that may not reflect natural conditions in the wild.

In *L. vulgaris*, postspawning egg care carries potentially high costs to adult specimens due to the increased exposure to predators while they are guarding the eggs. Additionally, males and females invest time and energy that could be used to copulate with other individuals. A hypothesis underlying this behavior could be that the female and/or male are the parents of some of the eggs, thus representing a parental care behavior. In this case, there may be significant selective advantages, such as avoiding egg predation in order to maximize the success of offprint survival (genetic material transmission; Partridge & Harvey, 1988). Furthermore, the presence of potential threats, such as divers, could trigger a heightened protective response, prompting the squid to return to the egg‐laying site to ensure the safety of its eggs.

Although there are similarities in reproductive behavior among several squid species, our observations of *L. vulgaris* indicate some degree of collaboration between males and females in egg guarding. Recently, Sampaio et al. (2021) addressed that male squid invest more in the transmission of their genes than previously believed. These authors reported that males of *Sepioteuthis lessoniana* collaborate in the search for a suitable location for the female to lay eggs. Since there are relatively few published studies of in situ egg‐laying activities, it is difficult to know what is normal and what altered behavior patterns are due to the presence of human observers, cameras, lights, etc. However, our finding is unexpected because this behavior differs from the reproductive habits assumed for European squid. Furthermore, it may be the first evidence of egg guarding by males in cephalopods, opening an interesting research line, not only on how this behavior affects fitness but also on its impact on evolution and gene transfer. Further observations and studies will help us to better understand the ecological and evolutionary drivers behind this behavior.

## AUTHOR CONTRIBUTIONS

**Jorge Hernández‐Urcera:** Conceptualization (equal); data curation (lead); formal analysis (lead); funding acquisition (supporting); investigation (equal); methodology (equal); software (lead); validation (lead); visualization (lead); writing – original draft (lead); writing – review and editing (equal). **Ángel F. González:** Conceptualization (equal); funding acquisition (lead); investigation (equal); project administration (lead); writing – review and editing (equal). **Felipe Escolano:** Conceptualization (equal); investigation (equal); methodology (equal); writing – review and editing (equal). **Miguel Cabanellas‐Reboredo:** Conceptualization (equal); data curation (equal); investigation (equal); methodology (equal); writing – review and editing (equal).

## FUNDING INFORMATION

J.H.‐U. and M.C.‐R. were supported by two of Juan de la Cierva's post doc research grants (IJC‐2020‐043701 and IJC‐2019‐038852, respectively; Ministerio de Ciencia e Innovación, Spain). ECOSUMA Project (PID2019‐110088RB‐I00) partially supported this study.

## CONFLICT OF INTEREST STATEMENT

The authors declare no competing interests.

## Supporting information


**Video S1:** Initial observation of egg masses and the first encounter with a male squid.

**Video S2:** Presence of both female and male squid in proximity to the egg masses.

**Video S3:** Interaction of female and male squid with the egg masses.

**Video S4:** Male squid (identical specimen recorded on the previous day) interacting with the egg masses alone.

**Video S5:** Observation of the first hatchlings.

## Data Availability

The original contributions presented in the study are included in the article or Supporting Information (Videos [Link], [Link]). Further inquiries can be directed to the corresponding author.
